# Postoperative Communicating Hydrocephalus Following Grade 2/3 Glioma Resection: Incidence, Timing and Risk Factors

**DOI:** 10.3390/cancers15143548

**Published:** 2023-07-09

**Authors:** Lisa S. Hönikl, Nicole Lange, Bernhard Meyer, Jens Gempt, Hanno S. Meyer

**Affiliations:** 1 Department of Neurosurgery, Klinikum Rechts der Isar, School of Medicine, Technical University of Munich, Ismaninger Str. 22, 81675 Munich, Germany; lisa.hoenikl@tum.de (L.S.H.); nicole.lange@tum.de (N.L.); bernhard.meyer@tum.de (B.M.); j.gempt@uke.de (J.G.); 2 Department of Neurosurgery, University Medical Center Hamburg-Eppendorf, Martinistraße 52, 20246 Hamburg, Germany

**Keywords:** glioma, hydrocephalus, cerebrospinal fluid shunt, risk factors, postoperative

## Abstract

**Simple Summary:**

Postoperative communicating hydrocephalus can develop following glioma resection. Especially in glioblastoma, this has been well documented. However, little is known about post-resection hydrocephalus in lower-grade gliomas. We analyzed a large cohort of patients who underwent lower-grade glioma resection to determine the frequency and timing of post-resection hydrocephalus and to identify risk factors. We found that higher tumor grade, postoperative re-bleeding, ventriculitis and infratentorial tumor localization are significant independent risk factors. Physicians treating brain tumor patients should be aware that postoperative communicating hydrocephalus requiring shunting occurs not only in glioblastoma but also after resection of lower-grade gliomas, especially in grade 3 tumors.

**Abstract:**

Background: In diffusely infiltrating gliomas, the maximum extent of tumor resection is an important predictor of overall survival, irrespective of histological or molecular subtype or tumor grade. For glioblastoma WHO grade 4 (GBM), it has been shown that resection-related events, such as ventricular opening and ventriculitis, increase the risk for development of communicating hydrocephalus (CH) requiring cerebrospinal fluid (CSF) diversion surgery. Risk factors for the development and the incidence of hydrocephalus following resection of other types of infiltrating gliomas are less well established. In this study, we evaluated the incidence and timing of occurrence of different types of hydrocephalus and potential risk factors for the development of CH following resection of grade 2 and 3 gliomas. Methods: 346 patients who underwent tumor resection (WHO grade 2: 42.2%; 3: 57.8%) at our department between 2006 and 2019 were analyzed retrospectively. For each patient, age, sex, WHO grade, histological type, IDH mutation and 1p/19q codeletion status, tumor localization, number of resections, rebleeding, ventriculitis, ventricular opening during resection and postoperative CSF leak were determined. Uni- as well as multivariate analyses were performed to identify associations with CH and independent risk factors. Results: 24 out of 346 (6.9%) patients needed CSF diversion surgery (implantation of a ventriculoperitoneal or ventriculoatrial shunt) following resection. Nineteen patients (5.5%) had CH, on median, 44 days after the last resection (interquartile range: 18–89 days). Two patients had obstructive hydrocephalus (OH), and three patients had other CSF circulation disorders. CH was more frequent in grade 3 compared to grade 2 gliomas (8.5 vs. 1.4%). WHO grade 3 (odds ratio (OR) 7.5, *p* = 0.00468), rebleeding (OR 5.0, *p* = 0.00984), ventriculitis (OR 4.1, *p* = 0.00463) and infratentorial tumor localization (OR 6.6, *p* = 0.00300) were identified as significant independent risk factors for the development of post-resection CH. Ventricular opening was significantly associated with CH, but it was not an independent risk factor. Conclusion: Physicians treating brain tumor patients should be aware that postoperative CH requiring CSF shunting occurs not only in GBM but also after resection of lower-grade gliomas, especially in grade 3 tumors. It usually occurs several weeks after resection. Rebleeding and postoperative ventriculitis are independent risk factors.

## 1. Introduction

Gliomas are the most common primary brain tumors. They can be classified by their histological subtype (such as glioblastoma, astrocytoma, oligodendroglioma and ependymoma), their molecular properties (e.g., IDH mutation and 1p/19q codeletion status) and by their grade of malignancy according to the World Health Organization (WHO) classification of tumors of the central nervous system [1,2,3]. The standard therapy of gliomas usually entails maximal safe tumor resection and, depending on the subtype, adjuvant radiation therapy and/or chemotherapy [4,5,6,7,8,9].

Glioblastoma (GBM) is the most common glioma type in adults. With one of the lowest survival rates of any neoplasm, it has been regarded as one of the deadliest of diseases [10]. Patients with diffusely infiltrating grade 2 or 3 gliomas have a better prognosis, but a definitive cure can usually be achieved in grade 1 gliomas only. However, evidence-based therapeutic regimes taking established molecular tumor subtypes into account and improved surgical strategies have led to a gradual increase in the overall survival of patients with lower-grade gliomas [5,6,7,11]. Consequently, more patients undergo multiple treatment cycles and resection surgeries, possibly leading to an increase in treatment-related complications and/or disease-related long-term complications, such as hydrocephalus.

Hydrocephalus, i.e., the abnormal accumulation of cerebrospinal fluid (CSF) within the ventricular system, can lead to massive symptoms associated with increased intracranial pressure, such as nausea, headache, cognitive decline and, eventually, death. The management of hydrocephalus includes external ventricular drainage and ventriculoperitoneal (VP) or ventriculoatrial (VA) shunting [12]. Hydrocephalus can be classified as communicating (CH) or obstructive (OH). While the former is thought to be a consequence of decreased absorption or, in rare cases, of overproduction, the latter is caused by physical obstruction [13,14]. In gliomas, OH can occur as a consequence of tumor growth, leading to an obstruction of the foramen of Monro, the third or fourth ventricle or the aquaeductus cerebri. In GBM, CH is typically seen in patients who have undergone therapy such as resection or radiation [15,16]; little is known about CH in lower-grade gliomas. In children with brainstem or posterior fossa gliomas, 80–90% have been reported to develop hydrocephalus [17,18]. In adults, the incidence of CH following surgery appears to be lower than 10% in GBM [15,16,19,20,21]. For lower-grade gliomas, this has not been established, and it is unclear whether the risk for postoperative CH in glioma patients is related to the WHO grade. Reported risk factors in GBM patients are ventricular opening during tumor resection [15], leptomeningeal tumor spread [16] and the number of pre-shunt craniotomies [22]. In a recent study, we found that 7% of GBM patients undergoing resection develop CH, and we identified ventricular opening, ventriculitis and CSF leak as independent risk factors for post-resection CH [21].

The aim of this study was to investigate the incidence and timing of occurrence of hydrocephalus and to identify risk factors for the development of CH requiring permanent CSF diversion following resection of diffusely infiltrating lower-grade (WHO 2/3) gliomas based on a retrospective analysis of all glioma resection cases at our institution between 2006 and 2019.

## 2. Materials and Methods

### 2.1. Study Design and Patient Selection

We retrospectively analyzed a prospectively collected database of 346 patients who underwent glioma resection at our institution between 2006 and 2019. All patients had histologically confirmed gliomas (WHO grades 2 or 3) [2]. Methods were equal to those of a previous study on hydrocephalus following resection of GBM [21].

### 2.2. Treatment

All patients underwent microsurgical resection aiming at maximal safe tumor removal based on the recommendation by an interdisciplinary tumor board. Standard procedure at our institution includes transcranial magnetic stimulation-based preoperative functional language and motor mapping and intraoperative neuromonitoring. The placement of an external ventricular drain following resection was not standard procedure, including cases with ventricular opening. Post-resection adjuvant treatment was based on tumor board recommendations and included radiation and/or chemotherapy if indicated according to current treatment standards [5,6,7,8,11].

### 2.3. Hydrocephalus

The diagnosis of hydrocephalus was based on radiographic assessment (CT and/or MRI showing an increase in ventricle size over the course of treatment that was not associated with atrophy and/or signs of CSF diapedesis) and clinical symptoms such as headache, cognitive impairment, urinary incontinence and/or papilledema. Hydrocephalus types were defined as communicating (i.e., CSF circulates freely within and can exit the ventricles) or non-communicating/obstructive (i.e., CSF is prevented from exiting the ventricles based on a mechanical obstruction of CSF flow, e.g., at the foramen of Monro, the aquaeductus cerebri and/or the fourth ventricle due to the mass effect of a tumor and/or its edema or to a blood clot). We also identified cases with other types of CSF circulation disorder (CSF entrapment within the resection cavity, subdural hygromas). In some cases, hydrocephalus was transient (e.g., obstructive hydrocephalus due to temporary resection-related edema). Acute hydrocephalus was typically treated with temporary placement of an external ventricular drain. Permanent hydrocephalus (i.e., CH, or OH with permanent obstruction) was treated with permanent CSF diversion surgery (ventriculoperitoneal or ventriculoatrial shunting). The indication for permanent CSF diversion surgery represents the event that determined whether a patient was regarded to have developed a type of hydrocephalus for the purposes of this study. 

### 2.4. Patient Characteristics

Our lower-grade glioma database comprises demographic and clinical parameters including sex, age, survival, number of tumor resections, adjuvant treatment modalities, tumor histology, molecular tumor properties, tumor location, ventricular opening during resection and postoperative complications such as ventriculitis, CSF leak and rebleeding. Ventriculitis was diagnosed based on laboratory analyses of CSF (elevated cell count, elevated lactate, low glucose) and/or positive CSF culture. Clinical signs and symptoms suggestive of ventriculitis leading to CSF analysis included fever, nuchal rigidity, neurological deterioration and/or reduced general condition unless otherwise explained. Rebleeding was defined as a postoperative bleeding seen on CT and/or MRI that required operative intervention. For the survival analysis, the date of death or, if unavailable, the last contact with the patient was used as endpoint.

### 2.5. Statistical Analysis

Statistical analyses were performed using R 3.2 (R Core Team). Associations between variables were analyzed using chi-squared tests. To identify independent risk factors for hydrocephalus, logistic regression analysis was performed. For all analyses, a difference with an error probability of less than 0.05 was considered statistically significant. Descriptive statistics for demographic variables were generated with means and standard deviations (SDs) or medians with interquartile ranges (IQRs) as appropriate. 

## 3. Results

### 3.1. Patient Characteristics

**Figure 1:**
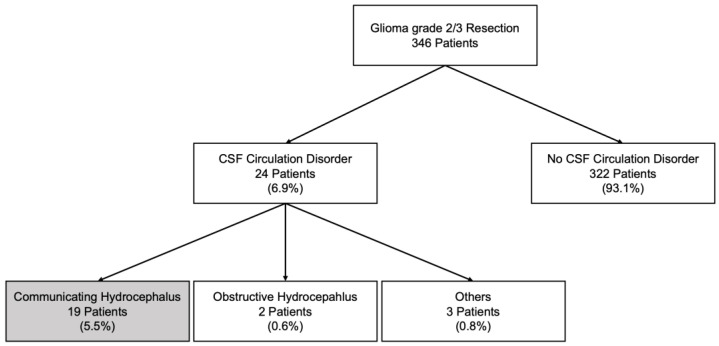
Incidence of different types of CSF circulation disorders in 346 glioma (WHO grades 2–3) patients who underwent tumor resection between 2006 and 2019. Twenty-four patients developed permanent postoperative CSF circulation disorders, corresponding to an incidence of 6.9%. Of these, 19 had communicating hydrocephalus (CH; 5.5%), 2 had obstructive hydrocephalus (OH; 0.6%) and 3 had other types of CSF circulation disorders (2 had hygroma and 1 had ventricular entrapment; 0.8%).

Our database contained 346 patients who underwent resection of a histologically identified grade 2 or 3 glioma at our institution between 2006 and 2019 (Figure 1). On average, patients were 38 years old (Table 1), and 59% were male. The majority had astrocytomas, followed by oligodendrogliomas and a few ependymomas. In total, 58% of tumors were grade 3. Information on IDH mutation status was available in 207 patients (81% had a mutation). Two-hundred thirteen cases had information on 1p/19q codeletion (40% had a codeletion). Most tumors were supratentorial, the majority of which were frontal and temporal.

### 3.2. The Incidence and Timing of Hydrocephalus after Glioma Resection

**Figure 2:**
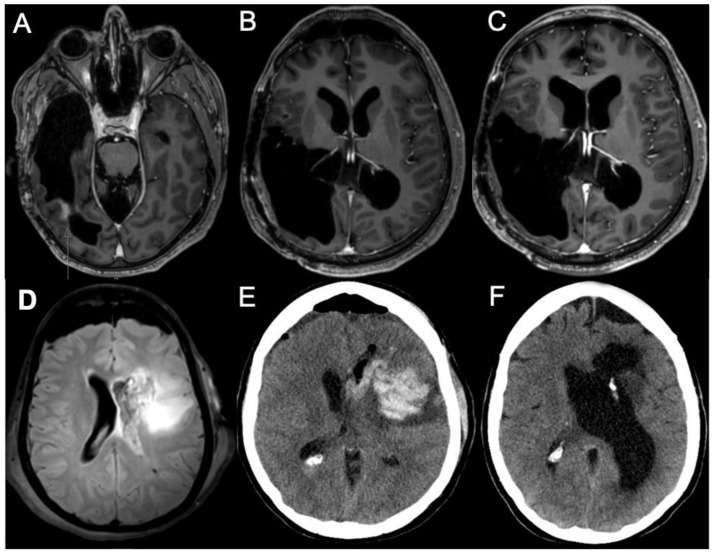
Case illustrations of two glioma patients who developed CH. Case 1 (**A**–**C**) was a 40-year-old male patient who underwent resection of a recurrent right temporal anaplastic oligodendroglioma ((**A**), preop MRI, t1 with contrast enhancement; (**B**), postop MRI). He had ventriculitis seven days after resection, and CH was diagnosed in the further course of the disease (**C**). Case 2 (**D**–**F**) was a 41-year-old male patient with a left frontal oligodendroglioma ((**D**), MRI showing left frontal FLAIR-hyperintense lesion). The tumor was removed, and the left lateral ventricle was opened. Some hours after surgery, a rebleeding occurred ((**E**), computed tomography (CT)). One month after revision surgery, CH was diagnosed with typical clinical presentation ((**F**), CT).

Twenty-four patients (6.9%) developed CSF circulation disorders requiring CSF diversion after tumor resection (Figure 1 and Figure 2, Table 1), most of which had CH (19 patients, 5.5%; for a comparison with the 322 resected patients who did not develop hydrocephalus, see Table 1). Five patients had other types of CSF circulation disorder (two had OH, one had resection cavity entrapment and two had subdural hygroma). The incidence of CH was much higher in grade 3 than in grade 2 tumors (8.5 vs. 1.4%). It was 3.9% in astrocytoma, 7.3% in oligodendroglioma and 14.2% in ependymoma (note, however, the relatively low number of ependymomas in our series; see Table 1). CH was more frequent in IDH-mutated gliomas (6.0 vs. 2.5%) and 1p/19q-codeleted gliomas (5.8 vs. 3.9%). With regards to tumor location, it was most frequent in infratentorial (15.4%) and multi-lobar supratentorial (7.7%) tumors. It occurred in 5.5% of frontal and 2.6% of temporal tumors. There were no parietal or occipital tumors that led to CH. CSF shunting for CH was, on average, performed 44 days (median) after the last resection surgery (OH: 23 days; entrapments/hygromas: 76 days; all types of hydrocephalus: 42 days). The time from resection to shunting for CH was variable (IQR: 18–89 days).

### 3.3. Risk Factors for the Development of CH after Glioma Resection

We investigated potential risk factors for the development of CH following glioma resection. Age (Spearman’s rho: 0.07; *p* = 0.103) and sex (*p* = 0.69) were not associated with CH, and neither were the histological subtype nor frontal (*p* = 0.29) or temporal (*p* = 0.31) tumor location (univariate analyses, see Table 2). Infratentorial tumor location, however, was identified as an independent risk factor (multivariate analysis, OR 6.6 [CI 1.8–22.5], *p* = 0.003, Table 2). IDH mutation (*p* = 0.226) and 1p/19q codeletion (*p* = 0.45) were also not associated with the development of CH. Apart from infratentorial location, the only patient-/tumor-related factor significantly associated with CH was tumor grade 3; it was also an independent risk factor (Table 2; OR 7.5 [2.2–38.2], *p* = 0.00468). 

We also investigated surgery-related risk factors (Table 1 and Table 2). Rebleeding had occurred in 6.5% of patients without hydrocephalus and in 15.8% of those who developed CH. Rebleeding was an independent significant risk factor for the development of postoperative CH (OR 5.0 [1.4–16.5], *p* = 0.00984,). On average (median), CSF shunting surgery was performed 29 (IQR: 24–104) days after rebleeding and 29 (24.5–105.5) days after the last tumor resection prior to shunting in these patients. 

Postoperative ventriculitis also turned out to be an independent risk factor (OR 4.1 [1.5–11.0], *p* = 0.00463). It was found in 10.9% of patients without hydrocephalus and in 42.1% of patients with CH. CSF shunting was performed 26 (IQR: 11–45) days after the onset of ventriculitis and 53 (IQR: 25.5–81.5) days after the last tumor resection. 

Ventricular opening was also significantly associated with CH (OR 4.7 [1.1–20.3], *p* = 0.039). It had occurred in 70.2% of patients without hydrocephalus and in 94.7% of those who developed CH. Time to CSF shunting was 72 (IQR: 40–794) days in this group. However, ventricular opening was not identified as an independent risk factor (*p* = 0.15499).

A postoperative CSF leak had occurred in 15.8% of patients with CH and in 8.1% of those without. CSF leak was not significantly associated with CH (*p* = 0.16).

### 3.4. Multiple Resections

On average (mean), glioma patients without hydrocephalus had 1.9 tumor resections (range 1–6), and those who developed CH had 2.5 (1–5) resections. Tumor re-resections were performed in 58% of patients without postoperative hydrocephalus and in 68% of patients who developed CH (Table 1). Having more than one resection was not associated with CH (*p* = 0.949, Table 2).

There were cases with multiple re-resections in patients without hydrocephalus and in patients with CH (one re-resection: 39.4% of all patients without hydrocephalus vs. 21.1% of all patients with CH; two re-resections: 13.0 vs. 21.1%; three re-resections: 3.7 vs. 21.1%; four re-resections: 1.6 vs. 5.3%; five re-resections: 0.3 vs. 0.0%). 

**Figure 3:**
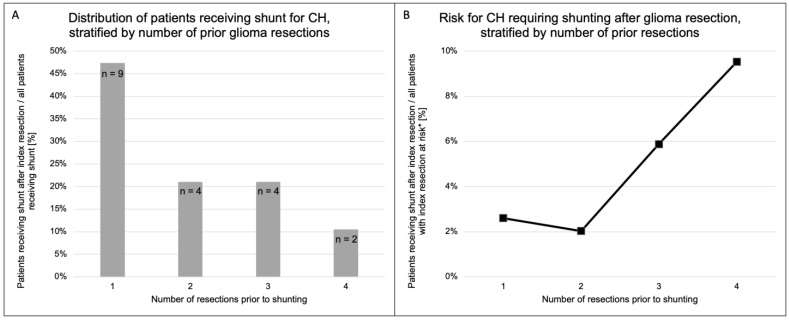
Relation of the number of glioma resections and shunting for CH. (**A**) Illustration of the relative frequencies of patients receiving a shunt for CH, stratified by the number of resection surgeries prior to shunting. Nearly 50% of the patients received their shunt after the first resection surgery. The risk for the development of CH after glioma resection increased in patients undergoing three and four resections (**B**). * “at risk” = patients who do not have a shunt already.

CH was diagnosed after the first resection surgery in half of the patients, and the other half occurred after re-resections (second to fourth resection, Figure 3A). The risk for the development of CH increased from less than 3% to 6% and almost 10% with the third and fourth resection (Figure 3B). 

Both rebleeding and ventriculitis, if present in patients with CH and multiple resections, occurred only once in the course of disease and mostly occurred following the last pre-shunting resection. Four out of five patients with CH and rebleeding had multiple resections. All those rebleedings had occurred within the context of the last pre-shunting resection surgery. Five out of eight patients with CH and ventriculitis had multiple resections. In four out of those five patients, ventriculitis had occurred within the context of the last pre-shunting resection surgery.

## 4. Discussion

### 4.1. The Incidence of Communicating Hydrocephalus after Glioma Resection

We present, to our knowledge, the first study investigating the incidence of post-resection hydrocephalus and risk factors for the development of CH in lower-grade gliomas. 

We found that 6.9% of glioma patients developed CSF circulation disorders requiring CSF diversion surgery in the course of their disease following resection; 5.5% had CH. This is a considerable number given that postoperative hydrocephalus can negatively affect the clinical course of glioma patients in multiple ways. It can lead, e.g., to slower or even permanently worse neurological recovery. Moreover, since we found that it occurs, on average, a few weeks after resection, it can also interfere with or delay adjuvant therapies.

Importantly, while the development of CH was a rare event in grade 2 gliomas (1,4%), it was much more frequent in grade 3 gliomas (8,5%). The latter suggests a similarity to GBM, as the incidence of CH following tumor resection in grade 3 tumors in this series is well within the range of previous studies on hydrocephalus in GBM that reported rates from 2.1 to 10% [15,16,19,21,23]; in our own series, 6.5% of GBM patients developed CH following resection [21].

In the brainstem and posterior fossa gliomas of children, the incidence of hydrocephalus has been reported as 80–90% [17,18]. Twenty-six patients in our cohort had infratentorial gliomas, and 19.2% of these had CSF diversion surgery. At our department, most of the pediatric cases are usually transferred to children’s hospitals after resection and lost to follow-up early. Given this and the fact that this is a retrospective study, we may underestimate the overall incidence of hydrocephalus, but especially in pediatric cases. 

### 4.2. Risk Factors for the Development of Communicating Hydrocephalus after Glioma Resection

The size of our series also enabled us to identify several independent risk factors for the development of post-resection CH in lower-grade glioma. Apart from infratentorial tumor location and WHO grade 3, all of them were surgery-related factors. 

Rebleeding was identified as an independent significant risk factor for the development of CH after glioma resection. In our cohort, 7.5% of patients had rebleeding, 11.5% of which developed CH. This is in line with several studies reporting that intracranial hemorrhage, such as subarachnoid or subdural hemorrhage, can lead to a disturbed CSF circulation [24,25,26]. In a large retrospective analysis of patients with aneurysmal subarachnoid hemorrhage, e.g., the prevalence of shunt-dependent hydrocephalus was 22.3%. Spontaneous intraparenchymal as well as intraventricular bleeding have been identified as independent significant risk factors as well. It can be assumed that rebleeding in the tumor resection cavity and/or the ventricles trigger similar pathophysiological mechanisms, such as agglutination of the arachnoid granulations and, eventually, communicating hydrocephalus [25]. Interestingly, even though ventricular opening was not an independent risk factor in this study, it was present in all but one of the cases that developed post-resection CH, suggesting that it was not a sufficient but a virtually mandatory condition for the development of CH. In our previous study on post-resection hydrocephalus in glioblastoma, we found that ventricular opening, but not rebleeding, was an independent risk factor [21]. This could actually support both being related to the development of hydrocephalus: it is likely that during a typical glioblastoma resection, there is more intraoperative bleeding/debris during resection, meaning that the presence or absence of ventricular opening is key for determining the risk of developing CH, consistent with previous studies [15,16,23]. In lower-grade gliomas, on the other hand, a typical resection produces less intraoperative bleeding that could enter the ventricles. If there was ventricular opening, however, a post-resection rebleeding would then trigger mechanisms similar to intraoperative debris-related CH following glioblastoma resection. At any rate, it appears to be reasonable to prevent ventricular opening during resection, if possible, or to cover the opening during resection to prevent debris from entering the ventricles.

We found that postoperative ventriculitis is another independent risk factor for post-resection CH in glioma, as we recently demonstrated for GBM as well [21]. In the present study, 12.7% of patients had postoperative ventriculitis, 18.2% of which developed CH. The rate of postoperative ventriculitis/meningitis in our series is consistent with the relatively high rate we found in our series on glioblastoma patients, but it is higher than in previous studies from other centers [27,28,29]. Of note, many patients in our cohort had multiple resections (and, accordingly, multiple lines of therapy), and it can be speculated that this contributed to the relatively high infection rate [30,31,32]. 

Our data suggest that the risk for post-resection CH increases with the number of tumor resections (Figure 3B). Due to the relatively low absolute number of patients with a high number of resections, this has to be interpreted with caution, but it would be consistent with studies claiming that the number of craniotomies plays a significant role in the development of post-resection CH [15] and our own series on post-resection CH in glioblastoma [21]. The putatively higher risk for post-resection CH should be kept in mind when discussing possible re-resections with the patient.

Our study was focused on surgery-related risk factors. Other possible risk factors were not the focus of this study, such as leptomeningeal or intraventricular tumor dissemination or radiation [19,33,34,35].

### 4.3. Study Limitations

This was a retrospective analysis, potentially leading to selection bias. Our results have to be interpreted based on circumstances specific to our department (e.g., patient collective, such as the fact that we treat mostly adults; or treatment standards, cf. Methods). We also may have lost patients undergoing CSF diversion surgery elsewhere, and the incidence of shunting for CH in our cohort could thus be regarded as a lower-bound estimate. 

Moreover, since our cohort spans more than a decade, molecular data were not available for all patients, meaning that not all tumor diagnoses could be made according to the latest WHO classification [35]. However, at least information on IDH mutation and 1p/19q co-deletion status was available for most patients. Different from WHO grade, there was no obvious relationship between these markers and the risk for post-resection CH. However, whether there is a diagnosis-specific risk beyond the WHO grade, which is clinically less relevant than established molecular markers, will have to be investigated in future studies that ideally rely on a prospective design.

## 5. Conclusions

Postoperative CH requiring shunting surgery occurs in diffuse gliomas of lower grades, with a higher risk in grade 3 tumors. Shunts are typically required several weeks after resection. Surgery-related events including rebleeding and ventriculitis increase the risk for the development of CH. The possibility of post-resection CH should be discussed both with the patient ahead of surgery and with subsequently treating physicians. 

## Figures and Tables

**Table 1:** Characteristics of glioma patients with and without different types of postoperative CSF circulation disorders, classified as communicative hydrocephalus (CH) and other CSF circulation disorder types (occlusive hydrocephalus, hygroma, resection cavity entrapment). CH was by far the most frequent type. Surgery-related factors are also reported.

	All	CSF Circulation Disorder		
		None	CH	Other
**N of patients (% of all)**	346	322 (93.1%)	19 (5.5%)	5 (1.4%)
**Median Age (years (min–max))**	38 (5–90)	38 (6–90)	38 (5–68)	74 (29–80)
**Male sex (*n* (%))**	203 (58.7%)	188 (58.4%)	11 (57.9%)	4 (80.0%)
**WHO Grade (*n* (% of column))**				
2	146 (42.2%)	143 (44.4%)	2 (10.5%)	1 (20.0%)
3	200 (57.8%)	179 (55.6%)	17 (89.5%)	4 (80.0%)
**Histology (*n* (% of column))**				
Astrocytoma	205 (59.2%)	195 (60.6%)	8 (42.1%)	2 (40.0%)
Oligodendroglioma	123 (35.5%)	112 (34.8%)	9 (47.4%)	2 (40.0%)
Ependymoma	14 (4.0%)	11 (3.4%)	2 (10.5%)	1 (20.0%)
Others *	4 (1.2%)	4 (1.2%)	0 (0.0%)	0 (0.0%)
Molecular Type (*n* (% **))				
IDH mutation	167 (80.7%)	153 (79.7%)	10 (90.9%)	4 (100.0%)
IDH wildtype	40 (19.3%)	39 (20.3%)	1 (9.1%)	0 (0.0%)
1p/19q deletion	86 (40.4%)	79 (39.7%)	5 (50.0%)	2 (50.0%)
1p/19q wildtype	127 (59.6%)	120 (60.3%)	5 (50.0%)	2 (50.0%)
**Tumor Localization (*n* (% of column))**				
Frontal	165 (47.7%)	153 (47.5%)	9 (47.4%)	3 (60.0%)
Temporal	77 (22.3%)	75 (23.3%)	2 (10.5%)	0 (0.0%)
Parietal	23 (6.6%)	23 (7.1%)	0 (0.0%)	0 (0.0%)
Occipital	3 (0.9%)	3 (0.9%)	0 (0.0%)	0 (0.0%)
More than one lobe	52 (15.0%)	47 (14.6%)	4 (21.1%)	1 (20.0%)
Infratentorial	26 (7.5%)	21 (6.5%)	4 (21.1%)	1 (20.0%)
**Surgical Factors (*n* (% of column))**				
>1 Tumor resection	201 (58.1%)	187 (58.1%)	13 (68.4%)	1 (20.0%)
Rebleeding	26 (7.5%)	21 (6.5%)	3 (15.8%)	2 (40.0%)
Ventriculitis	44 (12.7%)	35 (10.9%)	8 (42.1%)	1 (20.0%)
Ventricular opening	248 (71.7%)	226 (70.2%)	18 (94.7%)	4 (80.0%)
CSF Leak	30 (8.7%)	26 (8.1%)	3 (15.8%)	1 (20.0%)

* Includes mixed gliomas and gangliogliomas; ** % of all with IDH mutation or 1p/19q deletion status available.

**Table 2:** Overview of uni- and multivariate analyses of potential risk factors for the development of CH.

	Univariate		Multivariate	
	OR (CI)	*p*	OR (CI)	*p*
WHO grade 3	5.6 (1.6–19.1)	**0.0060**	7.5 (2.2–38.2)	**0.00468**
IDH mutation	3.6 (0.5–28.0)	0.2260		
1p/19q codeletion	1.5 (0.5–4.5)	0.4500		
Frontal tumor	1.6 (0.7–3.9)	0.2890		
Temporal tumor	0.6 (0.2–1.7)	0.3124		
Infratentorial tumor	3.8 (1.3–11.1)	**0.0160**	6.6 (1.8–22.5)	**0.00300**
>1 Tumor resection	1.0 (0.4–2.3)	0.9490		
Rebleeding	3.8 (1.3–11.1)	**0.0160**	5.0 (1.4–16.5)	**0.00984**
Ventriculitis	4.9 (2.1–12.1)	**0.0005**	4.1 (1.5–11.0)	**0.00463**
Ventricular opening	4.7 (1.1–20.3)	**0.0390**	3.0 (0.8–19.6)	0.15499
CSF Leak	2.3 (0.7–7.2)	0.1600		

Bold numbers indicate statistically significant risk factors.

## Data Availability

The data presented in this study can be shared upon reasonable request.
